# Stigma and epilepsy in onchocerciasis-endemic regions in Africa: a review and recommendations from the onchocerciasis-associated epilepsy working group

**DOI:** 10.1186/s40249-019-0544-6

**Published:** 2019-05-20

**Authors:** Sarah O’Neill, Julia Irani, Joseph Nelson Siewe Fodjo, Denis Nono, Catherine Abbo, Yasuaki Sato, Augustine Mugarura, Housseini Dolo, Maya Ronse, Alfred K. Njamnshi, Robert Colebunders

**Affiliations:** 1 CRISS – School of Public Health, Université Libre de Bruxelles and LAMC Faculté de Philosophie et de Sciences Sociales Université Libre de Bruxelles, Brussels, Belgium; 2 Department of Public health, Institute of Tropical Medicine, Antwerp, Belgium; 3 Global Health Institute, University of Antwerp, Antwerp, Belgium; 4 Department of Mental Health, Austrian Partnership Programme in Higher Education and Research for Development (APPEAR) Project & AVSI Foundation, Gulu University, Gulu, Uganda; 5 Department of Psychiatry, Makerere University, Kampala, Uganda; 6 Department of Environmental Science and Technology, Osaka Sangyo University, Osaka, Japan; 7 Epilepsy Support Association of Uganda, Wakiso, Uganda; 8 Department of Neurology, Yaoundé Central Hospital/FMBS, The University of Yaoundé I, Brain Research Africa Initiative (BRAIN), Yaoundé, Cameroon

**Keywords:** Epilepsy, Onchocerciasis, Stigma, Anti-epileptic treatment, Nodding syndrome, Discrimination, Misconception, Africa

## Abstract

**Background:**

In onchocerciasis-endemic areas, particularly in those with a sub-optimal onchocerciasis control programme, a high prevalence of epilepsy is observed. Both onchocerciasis and epilepsy are stigmatizing conditions. The first international workshop on onchocerciasis-associated epilepsy (OAE) was held in Antwerp, Belgium (12–14 October 2017) and during this meeting, an OAE alliance was established. In this paper, we review what is known about epilepsy-associated stigma in onchocerciasis-endemic regions, and present the recommendations of the OAE alliance working group on stigma.

**Main body:**

For this scoping review, literature searches were performed on the electronic databases PubMed, Scopus and Science Direct using the search terms “epilepsy AND onchocerciasis AND stigma”. Hand searches were also undertaken using Google Scholar, and in total seven papers were identified that addressed epilepsy-related stigma in an onchocercisasis-endemic area. Due to the limited number of published research papers on epilepsy-associated stigma in onchocerciasis-endemic areas, other relevant literature that describes important aspects related to stigma is discussed. The thematic presentation of this scoping review follows key insights on the barriers to alleviating the social consequences of stigma in highly affected onchocerciasis-endemic areas, which were established by experts during the working group on stigma and discrimination at the first international workshop on OAE. These themes are: knowledge gaps, perceived disease aetiology, access to education, marriage restrictions, psycho-social well-being, burden on the care-giver and treatment seeking behaviour. Based on the literature and expert discussions during the OAE working group on stigma, this paper describes important issues regarding epilepsy-related stigma in onchocerciasis-endemic regions and recommends interventions that are needed to reduce stigma and discrimination for the improvement of the psycho-social well-being of persons with epilepsy.

**Conclusions:**

Educating healthcare workers and communities about OAE, strengthening onchocerciasis elimination programs, decreasing the anti-epileptic treatment gap, improving the care of epilepsy-related injuries, and prioritising epilepsy research is the way forward to decreasing the stigma associated with epilepsy in onchocerciasis-endemic regions.

**Electronic supplementary material:**

The online version of this article (10.1186/s40249-019-0544-6) contains supplementary material, which is available to authorized users.

## Multilingual abstracts

Please see Additional file 1 for translations of the abstract into the five official working languages of the United Nations.

## Background

In onchocerciasis-endemic areas, particularly in those with a sub-optimal onchocerciasis elimination programme, a high prevalence of epilepsy is observed [1–4]. Onchocerciasis is caused by the nematode *Onchocerca volvulus*, which is transmitted by blackflies that breed along fast-flowing rivers. Besides causing skin lesions and eye disease resulting in blindness if not treated, onchocerciasis can also cause epilepsy [5], although the exact physiopathology of the latter is not yet known [1, 6–10]. Onchocerciasis-associated epilepsy (OAE), including nodding syndrome (NS – head nodding seizures, stunting, and cognitive decline [11–16] that have been associated with onchocerciasis), is characterized by seizures that start during childhood and adolescence, and is often associated with cognitive, behavioural and psychiatric problems [11, 16, 17]. A recent estimation of the OAE burden revealed that about 381 000 persons are currently affected [18]. If OAE remains untreated, severe physical and functional deficits may occur [4, 11, 14, 17]. The clinical features of onchocercal skin disease (OSD) include itching, papular and papulo-macular rash, skin atrophy and depigmentation [19]. While epilepsy and OSD have independently been associated with stigma [19–28], little is known concerning the effect of both epilepsy and onchocerciasis on the quality of life, psycho-social and economic well-being of affected persons. Furthermore, in many onchocerciasis-endemic areas, majority of persons with epilepsy (PWE) do not have access to appropriate anti-epileptic medication; this high proportion of untreated PWE constitutes a wide anti-epileptic treatment gap which exacerbates the symptoms and leads to the discrimination of affected individuals [24–26]. It is important to recognize and act upon the complexity and variability of the socio-cultural conceptions of epilepsy [27, 28], which contribute to stigma, both across and within affected communities since improved prevention and treatment strategies are urgently needed.

Stigma is a process whereby a person is disqualified from full social acceptance within a community or society because of a particular attribute they possess. Goffman (1964) first described and classified stigma as due to particular attributes such as: (i) visible physical deformation (i.e. scars or missing limbs); (ii) personal traits or lifestyles that deviate from what is perceived as the social norm (i.e. drug addiction, homosexuality, criminal background); (iii) “tribal stigmas” linked to ethnicity, nationality or religion [29]. The stigmatizing attribute is often accompanied by stereotypes that characterize its possessor in negative ways, such as “very dangerous”, “very contagious”, “unreliable”, “possessed” or “immoral” [29]. Link and Phelan (2001) described and conceptualized the way in which stigma develops through a sequential process: first by distinguishing or labelling a trait of human difference; this is followed by attaching a negative stereotype to the label. The third step is discrimination – separating “them” from “us”; finally, labelled persons experience loss of status and are considered inferior [30]. With regard to neurological diseases in the tropics, Tabah et al. (2014) argue that loss of status and discrimination are embedded in social, economic and political processes and grow in relevance and scope over time [23]. This growth of loss of status occurs as the negative stereotypes gradually become accepted and internalized by the community and by stigmatized individuals [23]. In many contexts, epilepsy is a stigmatizing condition for those affected and for their families [31–33]. The extent of the stigmatization can vary between contexts and can also be affected by an individual’s pre-morbidities, clinical course, drug side-effects and socio-demographic characteristics such as gender, education, employment status and social rank. Societal conceptualizations of the condition (i.e. tendency to blame versus sympathize with the sufferer) impacts upon the support services available to those affected [34–38]. Nevertheless, stigmatization is a dynamic process that can be aggravated, but may also decrease with time depending on changes in social norms and attitudes towards the condition [23]. Understanding the process of stigmatization is important in planning and implementing interventions to avert stigma [23, 27, 29]. During the first international workshop on OAE which held in Antwerp, Belgium from 12 to 14 October 2017 [39], an OAE alliance was established to engage multidisciplinary teams in OAE care and advocacy. In this paper, we review what is known concerning epilepsy-associated stigma in onchocerciasis-endemic regions and present the recommendations of the OAE alliance working group on stigma.

## Methods

The need for this scoping review was established during the discussions of the working group on stigma and discrimination at the international OAE workshop. Literature searches were performed on the electronic databases PubMed, Scopus and Science Direct. We limited the scope of this review to sub-Saharan Africa, because of high ongoing transmission of onchocerciasis in this part of the world in contrast to South America. Included were research papers citing empirical data and describing the phenomenon of stigma in onchocerciasis endemic areas. Using the search term “epilepsy AND onchocerciasis AND stigma”, five papers were identified in PubMed, four in Scopus and 18 in Science Direct. However, after careful screening of these papers, only three research papers were identified that addressed epilepsy-related stigma in an onchocercisasis-endemic area [24, 40, 41]. Hand searches through expert recommendations and searches on Google Scholar further identified four other relevant papers investigating stigma in a population affected by OAE [42–45]. The thematic presentation of this scoping review follows the key insights on the barriers to alleviating the social consequences of stigma in highly affected onchocerciasis-endemic areas, which were established by experts during the working group on stigma and discrimination at the first international workshop on OAE. These themes are knowledge gaps, perceived disease aetiology, access to education, marriage restrictions, psycho-social well-being, burden on the care-giver and treatment seeking behaviour. Due to the limited number of published research papers on epilepsy-associated stigma in onchocerciasis-endemic areas, we also cite other relevant literature that describes important aspects related to stigma i.e. medical professionals’ attitudes towards PWE. Furthermore, we report on relevant field research and clinical experiences of members of the OAE alliance working group, the social scientists (SO, JI, ND, CA, HD, YS, MR) and the clinicians (JNSF, AKN, RC) who all have been working in onchocerciasis-endemic regions. These insights contribute to a better understanding of how stigma affects PWE specifically in onchocerciasis-endemic areas.

## Main text

### What is different about epilepsy in onchocerciasis-endemic areas compared with non-endemic areas?

In sub-Saharan Africa, the mean epilepsy prevalence was found to be 9.39 per 1000 [46]. In onchocerciasis-hyperendemic regions, significantly higher epilepsy prevalences have been reported [1–3, 47–49]. In a recent cohort study, the risk of developing epilepsy was shown to increase proportionately with the intensity of infection with *O. volvulus* [5]*.* The peculiarities as regards epilepsy stigma in such settings are numerous: (i) OSD, which affects several PWE [50] is a stigmatizing condition; (ii) onchocerciasis-endemic villages are often remote, poorly accessible, with weak healthcare systems and wide antiepileptic treatment gaps; (iii) clustering of PWE in families and/or villages is frequent [2, 49, 51]; (iv) PWE, in particular those suffering from NS or Nakalanga syndrome, are often cognitively impaired, and may present with psychiatric disorders, stunting, facial dysmorphia, thoracic and spinal deformities [52]. Consequently, not only PWE but entire families and sometimes villages are avoided and subject to stigmatization [26, 53, 54]. As with epilepsy, people affected by OSD often suffer from poor self-esteem [19, 23] and women in particular have difficulties in finding a marriage partner [55]. In 2009, a study to evaluate the effect of implementation of community-directed treatment with ivermectin (CDTI) on onchocerciasis-related stigmatization, suggested that 7–10 years of CDTI improved the relationships between healthy people and those with OSD; however stigmatization persisted and people still feared sexual intimacy with affected persons [56]. In the past, people with OSD were considered unclean and stigmatized because of fear of OSD transmission and the shame associated with the condition. People who had lived in the community for less than 5 years, tended to stigmatize OSD patients more than those who had lived in the community for longer than 5 years [56]. This could be attributed to better awareness and familiarity with the condition.

An additional observation relative to epilepsy in onchocerciasis-endemic areas is that PWE with severe cognitive decline (e.g.children with NS) are sometimes tied to a tree using a rope to prevent them from wandering and getting lost when the family goes farming. Such practices further contribute to the stigmatization of these individuals and their families.

Despite the suffering and rejection experienced by PWE in onchocerciasis-endemic areas, many policy-makers and non-governmental organizations are not yet aware that onchocerciasis elimination efforts have the potential to prevent new cases of epilepsy from appearing [8]; whereas this has been demonstrated by the age shift among PWE following 19 years of CDTI in Cameroon [57] and by the drastic decrease in epilepsy incidence (including NS) observed in Uganda after strengthening onchocerciasis elimination strategies [8]. At present, public health and community measures addressing OAE are not put in place. The fact that community members and local healthcare workers in affected villages are not informed about onchocerciasis as the likely cause of the high prevalence of epilepsy in their village leads to uncertainty, fear, misconceptions and stigma towards PWE. Indeed, ignorance regarding the possible causes of epilepsy can foster discrimination and stigma as highlighted by Kaddumukasa et al. [58].

On the other hand, because of the high epilepsy prevalence in these settings, epilepsy is generally a well known condition and therefore there is a high demand for anti-epileptic drug treatment because the population has experienced that traditional medicine does not work [40].

### Epistemological conundrums and knowledge gaps

The cause, clinical presentation and case definition of NS, has been subject to extensive discussion within the scientific community [59]. While scientists are trying to work out the cause of the disease, community members have also developed illness explanatory models which are often linked to spiritual beliefs and social ills, based on local cosmologies and socio-cultural contexts. Despite the growing body of scientific literature on NS, there is a limited understanding of how community members perceive illness aetiologies across the different affected countries. Van Bemmel reports on the epistemological differences between the growing scientific evidence on epilepsy, which differ to some extent across countries, in contrast to how those directly affected actually understand and conceptualize the disease [59–62]. It is crucial to be aware of these epistemological differences and address knowledge gaps in interventions. Those affected evidently make sense of the condition based on their unique experiences and knowledge which may not include the most recent scientific discoveries.

### Perceived disease aetiology: evil afflictions and fear of infection

In many societies in sub-Saharan Africa, epilepsy is not perceived as a condition that is caused by a brain disorder but rather, that seizures are caused by malignant forces that might be associated with greed, sin or demonic possession [28, 43, 44, 63–68] Thus, at the community level, seizure attacks are met with fright and disbelief and are often thought to be a bad omen. One consequence therefore is avoidance due to fear. PWE are often subject to social exclusion and lack of contact with different members of the community and/or family [40, 42, 63–65]. Even healthcare workers, medical students and young physicians frequently have negative attitudes towards epilepsy [68, 69]. A convulsing person’s saliva, urine, breath and flatulence are often thought to carry infectious agents [24, 65, 70–74]. Fear of contagion may result in bystanders not intervening when PWE are having seizures to prevent injury. Profound psychological and physical disability may result, including burns [16, 17, 23, 44]. In the Mahenge area, an onchocerciasis-endemic region in Tanzania, Jilek-Aall reported that “among Wapogoro tribe it was widely held that touching a person with epilepsy during his seizure was very dangerous because the spirit could then leap over to the other person”. Furthermore she describes how a person affected by “epilepsy were given a deserted hut or was requested to build one for himself” [43]. In an onchocerciasis-endemic region of Liberia (West Africa) in 1983, a high epilepsy prevalence was observed (5%); Gerrits describes local Bassa and Kpelle beliefs of epilepsy and head nodding as being caused by the spirits of the dead (*ginna*) and water spirits (*mame wata*) [45]. As in many places, fear of contagion through saliva leads to the stigmatization, isolation and social exclusion of PWE [45]. The following is an example from northern Uganda, where a NS epidemic started during the Lord Resistance Army conflict around 2000, and shows that social exclusion and stigmatizing behaviour can be the result of fear of infection [62]. *“Most people in the community do not want to associate themselves with PWE because they think epilepsy is contagious. They fear contracting the illness. They cannot eat the same meal from the same bowl with PWE. Society do not want to associate with families with PWE”* [75]*.*

However, the extent of felt stigma and the ways in which epilepsy incites stigmatizing behaviour may differ in different settings. Some people in certain onchocerciasis-endemic villages with very high prevalence of epilepsy in northern Uganda react differently. They say, “*this disease can affect anybody. We don’t think it is contagious, because I care for my child and I am still ok. And utensils, food and sleeping areas are shared.”* [JI – ethnographic fieldwork Uganda 2015–2017]. Thus in some places where OAE is widespread, epilepsy is a common condition and, perhaps as a result of awareness-raising and accessibility to information about epilepsy, stigma is less severe.

Nevertheless, stigma may develop as a result of other aspects related to epilepsy – defecating and/or urinating on oneself, soiling the home, the bedding, food, as well as the extreme changes in physical appearance (drooling, burns, protruding teeth, stunting, etc) and cognitive decline [42]. These kinds of behaviours may thus also lead to avoidance by other community members and segregation.

### The social consequences of stigma

Observations in onchocerciasis-endemic areas show that PWE and their carers are often excluded from participating at community gatherings, wedding ceremonies and other traditional events or family celebrations. Some families are ashamed to be accompanied by their children with epilepsy. Social consequences affect the psycho-social well-being of PWE and their carers.

#### Access to education

Many children with epilepsy drop out of school at an early age because of poorly controlled epileptic seizures, cognitive impairments and stigma [24, 76, 77]. In Aketi, Bas Uélé province in the Democratic Republic of Congo (DRC), during focus group discussions with community members, it was mentioned that “children with epilepsy should not go to school because they could contaminate other children” [41]. Furthermore, the psychiatric manifestations may foster the belief that the child is possessed by an evil spirit and/or lead to humiliation or avoidance of the PWE by fellow pupils as well as teachers. As most schools are not prepared to receive children with epilepsy and teachers are rarely trained on how to deal with seizures, children often have to discontinue their education as teachers advise parents not to send them to school for “safety reasons” [70, 76, 78, 79]. Children with epilepsy who drop out of school become solely dependent on their caregivers, due in part to the poorly controlled epileptic seizures, cognitive impairments and stigma [34, 35, 76, 78, 79]. It has also been observed that non-affected siblings sometimes are compelled to drop out of school to compensate for the economic burden on the household and/or the caretaking of the affected child [24].

#### Marriage restrictions

Epilepsy is commonly thought to be hereditary [46, 64, 65]; this belief is even stronger in onchocerciasis-endemic areas, because of frequent clustering of PWE within the same household or village [2, 50, 52]. In some places, there are marriage restrictions regarding PWE for fear that the condition would affect the children. Although there is limited literature on the subject, in many communities across sub-Saharan Africa, PWE are often married by community members who are of lower social status. In places where marriages are arranged between particular families, epilepsy may break the ties of marriage obligation [43, 44]. Marriage opportunities with well-to-do or high social status families are limited [43, 44, 80–83]. In interviews with healthy adolescents in 12 villages of the Akwaya Health District, Cameroon, 68.7% would object to marrying a PWE [64]. Women with epilepsy frequently remain single [84], but have children whose fathers deny any association with the mother and the children. Men with epilepsy may also remain single or their wives leave them because they are not able to financially sustain a family [AKN and SO – observation 2016, Cameroon]. These social consequences affect the economic and psycho-social wellbeing of PWE and their families.

#### Psycho-social well-being

Uncontrolled seizures, mental and physical disabilities have a substantial impact on the quality of life as they can influence self-esteem, social relationships as well as physical well-being of both the caregivers and PWE [34, 43, 85]. As a result of social exclusion and being denied opportunities that apparently ‘healthy’ members of the community enjoy, many PWE, as well as their caregivers, suffer from feelings of self-blame and shame. Often little attention is paid to their pleas especially when epilepsy is perceived to be the punishment for wrong behaviour. Furthermore, uncontrolled epilepsy may cause mental debilitation in some PWE resulting in difficulties communicating with other community members. In addition, they may believe that they are not capable or not entitled to do things that ‘healthy’ members of the community do, as a result of an inferiority complex, social exclusion/protection or fear to “spread epilepsy”. Some PWE resort to violence to some extent triggered by frustration due to social exclusion and denial of social status, but also because of the brain damage associated with the epilepsy [14, 17]. At times, PWE get into fights or destroy the property of neighbours, which creates tensions between community members and sometimes leads to witchcraft accusations. In northern Uganda and other places in sub-Saharan Africa, some PWE (especially in cases of NS) are confined while the household members go out working. This is done to prevent the PWE from wandering about, potentially hurting themselves or causing social conflicts [24]. Similar situations have been described in the DRC:

*“People look at them with disrespect, and they speak with disregard. They say that the child there is crazy. I have been told myself that ‘your child is a mad man, you must not let him walk among the people’. While this disease makes them walk here and there like crazy. It disturbs their intelligence”* [40]*.*

#### Burden on the caregiver

For a child affected by OAE, functional independence is related to the impact of chronic stress on activities of daily living, including personal care, mobility and communication. One of the main challenges for the caregiver is to manage the child’s chronic health problems effectively and juggle this role with the requirements of everyday living [24]. Consequently, the task of caring for a child with complex disabilities like NS becomes daunting and emotionally draining for caregivers. The provision of such care may prove detrimental to both the physical health and the psychological well-being of caregivers of children with OAE. Besides caring for affected children, parents and caregivers have to deal with the extra burden of handling these challenging situations due to stigma and witchcraft accusations coupled with financial problems and managing the expectations from other members of the community. Children with epilepsy often cannot participate in income-generating activities and as a consequence, affected households with a poor support network spiral deeper into poverty [24, 42]. OAE is clustered not only in villages but also, within households [51]. With each child affected, there is a direct added cost in terms of treatment and medical care that includes transportation, caretaker’s time away from subsistence activities, as well as an increased need for personal hygiene products like soap, bedding and clothing. During an ethnographic study in northern Uganda, mothers often reported the need for sanitary napkins for their daughters as they were unable to clean their own clothes due to their condition and it was difficult for the mother to keep up with the washing (JI – ethnographic fieldwork 2015–2017). Community members also reported that females with epilepsy were often subjected to sexual abuse with a risk of acquiring sexually transmitted infections and if pregnant, left with the added burden of caring for the child themselves or by other female relatives, without the support of the father (JI – ethnographic fieldwork 2015–2017).

### Treatment-seeking behaviour for epilepsy in onchocerciasis endemic areas

Community perceptions of the condition affect the health seeking behaviour of those affected. As epilepsy is frequently linked to evil spirits/witchcraft, PWE and their families often seek remedies from traditional healers outside the hospital setting at some point in their health-seeking itinerary [28, 45, 63, 67]. Spiritual support can be psychologically comforting and meaningful for PWE and their families, and collaboration with traditional healers can be a positive experience [63]. However, some caregivers are also disillusioned and embittered about seeking remedies from spiritual guides and other kinds of traditional healers without the patient’s condition improving. The following quote from community leaders in DRC demonstrates the perceived lack of treatment options:

*“The medicine against epilepsy? There is not any that has been discovered to this moment. When you bring PWE to the traditional healers, they deceive us and then later, the seizure comes again, it is not good. If you have the drugs bring them to us”* [40]*.*

In some settings, parents and/or caregivers delay seeking biomedical advice until the PWE is severely affected and the treatments of a traditional healer have shown no improvement of the condition. Recourse to traditional treatments is particularly wide-spread in remote areas where there are limited and poorly resourced health-services and where there is no access to anti-epileptic drugs (AED) or where health centres regularly run out of stock. People’s interpretations of the cause of epilepsy become even more complex when no clear diagnosis or cure is ascertained. Due to the fact that PWE frequently interrupt AED because of irregular supply, lack of medical follow-up or for financial reasons, they may not experience the benefit of AED in terms of seizure control and therefore may not return to the health centre. Research in Cameroon shows that traditional healers are willing to co-operate with medical doctors and collaboration may be fruitful [63, 82]. Although the beneficial role of traditional healers in epilepsy care is still subject to debate, their involvement in epilepsy-related interventions especially in onchocerciasis-endemic areas warrants more research [21, 86].

## Discussion

Our review of the existing literature shows that onchocerciasis and epilepsy entail important social consequences linked to stigma and discrimination. There is voluminous literature describing epilepsy-related stigma [21, 23, 28]. Nevertheless, in terms of organized interventions addressing the social consequences of the condition, little has changed in onchocerciasis-endemic areas where people are more affected by co-morbidities, limited access to healthcare, lack of infrastructure, and higher levels of poverty. Remote rural villages with a high epilepsy prevalence require particular attention. Vast areas in the DRC, Tanzania and South Sudan, and most likely also the Central African Republic have not been sufficiently covered - neither in terms of onchocerciasis elimination efforts nor in terms of services that offer appropriate and uninterrupted symptomatic treatment of epilepsy [8, 43, 87].

Given the scarcity of stigma reduction interventions in the domain of epilepsy [58], we propose the following strategies to combat epilepsy stigma and the associated psycho-social consequences (Table 1). These are applicable in most settings; however in Table 1 we focus on intervention of particular importance in onchocerciasis-endemic areas.

**Table 1:** Measures and interventions to decrease epilepsy stigma

Recommended Intervention	Aim	How?
Epilepsy awareness campaign, with emphasis on OAE	Improve knowledge, decrease stigma	- Organize information campaign: locally, nationally, and internationally - Main message: Epilepsy is a non-contagious brain disease, which can be treated (not cured) - In onchocerciasis hyperendemic settings, > 85% of epilepsies are potentially caused by *O. volvulus* [5, 50, 52] and can therefore be prevented by adhering to CDTI.
Reduce epilepsy treatment gap	Access to uninterrupted supply of affordable AED	- Decentralize treatment/care services for PWE - Decrease stigma by improving knowledge to stimulate positive health-seeking behavior
Reducing knowledge gap about treatment	Improve adherence to AED treatment	- Message: AED treatment should not be interrupted - Discourage treatment using traditional medicine
Support for affected families	Improve their quality of life	- Provide psycho-social support for PWE and their families - Organize income-generating activities
Training healthcare workers (HCW)	Improve quality of care for PWE	- Set up comprehensive programs to train local HCW for the treatment, care, follow-up and co-morbidity management of PWE [50]
Training of teachers	Decrease school drop-out rates	- Set up training programs for teachers on how to deal with children with epilepsy
Support for community-based associations of PWE	Advocacy to improve the quality of life of PWE and their families	- Involve peer support groups - Use role models - Provide support for associations of PWE
Strengthening mental/brain health services and onchocerciasis elimination program	Early diagnosis and initiation of AED treatment. Decrease *O. volvulus* transmission to prevent new cases of OAE	- Increase collaboration between both programmes - Set up surveillance system for epilepsy - Monitor the quality of the services for PWE - Improve adherence with CDTI, stressing the importance of taking ivermectin to prevent OAE - Bi-annual CDTI in situations of high exposure to infected blackflies.
Strengthening the legal framework	Protect PWE against discrimination, abuse, rape	- Provide legal support for PWE
Cost of epilepsy studies	Evaluate the cost-effectiveness of interventions	- Conduct cost-effectiveness studies for epilepsy management in onchocerciasis-endemic areas with high epilepsy prevalence, prior to interventions
Fundraising	Increase funding for the treatment and prevention of epilepsy in onchocerciasis-endemic regions	- Establish OAE advocacy strategy - Create awareness about OAE among pharmaceutical companies, NGOs, international funding agencies - Document OAE burden of disease - Dissemination of OAE research findings - Obtain support from WHO, MOH, ILAE

*AED* Anti-epileptic drugs, *PWE* Person(s) with epilepsy, *CDTI* Community-directed treatment with ivermectin, *OAE* Onchocerciasis-associated epilepsy, *NGOs* Non-governmental organizations, *WHO* World Health Organizition, *MOH* Ministry of Health, *ILAE* International league against epilepsy

### Awareness campaigns

Awareness campaigns should explain the association of epilepsy with onchocerciasis infected blackflies and that the uptake of ivermectin reduces the occurrence of onchocerciasis and epilepsy. Such information campaigns have to be tailored to the public at local, national and international level. The message should be that OAE is preventable and treatable but not curable. Once people understand that PWE are not contagious and that their condition can be controlled and potentially prevented, it is likely that understanding the cause of OAE will reduce stigma.

### Reducing the epilepsy treatment gap

Reducing the epilepsy treatment gap could significantly improve the quality of life of PWE by potentially reducing seizures, which would reduce stigmatization, improve the health-conditions and decrease the risk of co-morbidities. Uninterrupted access to affordable AED is urgently needed to improve people’s quality of life and the psycho-social issues linked to the condition. To improve access to AED, the decentralization of treatment and care services is crucial [50]. There are current efforts to refine onchocerciasis endemicity maps through the World Health organization (WHO) and other neglected tropical disease (NTD) implementing partners. It is advisable to collaborate with these institutions to identify areas of high OAE prevalence. Engaging WHO should therefore be one of the priorities in addressing OAE.

### Reducing the knowledge gap

PWE on AED treatment need to understand that their condition requires regular follow-up and that they should not interrupt or change the dosage of their treatment without consultating a trained healthcare worker. Reducing the knowledge and treatment gaps will reduce stigma. A similar phenomenon has been observed with HIV infection. Once optimal antiretroviral drugs became widely available, a decline in social exclusion and stigmatization towards persons living with HIV infection was observed in some places [88]. In order to close the AED treatment gap, uninterrupted access to AED at the primary health care level is needed as interruption of AED can cause severe forms of seizures that can become fatal.

### Training of first-line healthcare workers and teachers

Research shows that medical staff and students display stigmatizing behavior and avoidance of people with epilepsy. Medical staff represent role models in the community; they are well respected as people trust their advice on prevention and treatment. If medical staff are seen showing avoidance behaviour and do not support a person who is having a seizure due to fear of contagion, then others are likely to copy their behaviour. The training of medical staff urgently needs to be re-inforced so that they understand that epilepsy is not contagious.

Teachers who are not trained on how to handle children with seizures can also play a crucial role in the stigmatization of people with epilepsy and their families in the community. Children with epilepsy are often asked not to come to class, sometimes because they do not know or do not feel competent to support a child having an epileptic seizure. Education is a human right and children with epilepsy should not be excluded from school. Teachers are also role models; their knowledge is respected and their behaviour influential on community perceptions. It is paramount that teachers should be better informed about the cause of epilepsy and how to deal with seizures that occur in the classroom. Ideally, in addition to teachers and health personnel, the entire community should receive basic instructions as to providing assistance to a PWE in the event of a seizure.

### Support for community-based associations of PWE

Community-based associations can help parents and caregivers support each other, engage in income-generating activities to decrease the economic burden as well as disseminating constructive evidence-based information about epilepsy. In support groups for PWE, people can learn from one another, share experiences, support one another and raise awareness about their problems. Peer support group attendance was shown to decrease internalized epilepsy-associated stigma among PWE in low-resource settings [89].

**Positive role models** are useful particularly for PWE whose seizures are not controlled. During the OAE workshop, participants emphasized that it is incredibly helpful for those in desperate situations to have positive examples of others whose epilepsy is controlled and who are able to live normal lives and participate in everyday activities. PWE representatives should be part of social group meetings and participate in lobbying for better treatment and care.

### Strengthening mental/brain health services and onchocerciasis elimination program

At present there are few infrastructures in place that systematically record data on epilepsy in conjunction with onchocerciasis. The quality of services for PWE needs to be monitored and improved. This will reduce epilepsy related morbidity, mortality and stigma and improve people’s psychosocial condition and quality of life. Recently, it was shown that many years of annual CDTI with insufficient ivermectin coverage did not result in an important decrease in prevalence and incidence of OAE [41, 90]. Therefore, to prevent children from developing OAE, bi-annual CDTI with optimal is needed in situations of high exposure to infected blackflies.

### Strengthening the legal framework

At present, the legal repercussions for discriminating behaviour against people with epilepsy are limited. Although research output is still limited, there is both anecdotal and research evidence suggesting higher rates of physical, psychological and sexual violence towards people with epilepsy [21]. Legal assistance and social support services are urgently needed to help victims fight for their rights. The local leadership (village headmen, area chiefs) need to be mobilised to develop local solutions (e.g village by-laws) for punishing discriminatory behaviours against PWE. A reduction of violence and increased legal support will reduce stigma.

### Fundraising

In order to implement these interventions increased advocacy is needed to put this major but neglected public health problem caused by epilepsy in onchocerciasis endemic regions on the international agenda of developing aid.

### Research priorities

#### Sexual violence and exploitation

Research on violence towards PWE and especially sexual violence and the sexual exploitation of women with epilepsy should be a research priority as literature is still scarce on the subject, although the phenomenon is commonly known to clinicians and social workers across sub-Saharan Africa. Sexual violence directly places the burden on women, who may get pregnant often with no support from the father. Sexual violence towards boys is also under-researched. As these topics are very sensitive an ethnographic approach is required.

#### Traditional healers

Traditional healers and their role in the reduction of epilepsy stigma needs to be explored further to fill knowledge gaps for interventions [86].

#### Measures and interventions to decrease epilepsy related stigma

Existing measures and future interventions that aim to reduce stigma and psycho-social problems as well as the burden of disease need to be evaluated. This will help improve existing infastructrures and provide evidence on what works and what doesn’t work.

## Conclusion

Persons with OAE carry a dual burden as both onchocerciasis and epilepsy are stigmatizing diseases. Providing information about the association between onchocerciasis and epilepsy, strengthening onchocerciasis elimination programmes, decreasing the anti-epileptic treatment gap, and improving the care of epilepsy-related complications is the way forward to decrease the stigma associated with epilepsy and to improve the quality of life of PWE and their families in onchocerciasis-endemic regions.

## Additional file

Additional file 1: Multilingual abstracts in the five official working languages of the United Nations. (PDF 534 kb)

## Abbreviations

AED: Anti-epileptic drugs
CDTI: Community-directed treatment with ivermectin
DRC: Democratic Republic of Congo
NTD: Neglected tropical disease
OAE: Onchocerciasis-associated epilepsy
OSD: Onchocercal skin disease
PWE: Person(s) with epilepsy
WHO: World Health Organization
