# Supplementary material for: Stigma and epilepsy in onchocerciasis-endemic regions in Africa: a review and recommendations from the onchocerciasis-associated epilepsy working group
Source: Infect Dis Poverty. 2019 May 20;8:34. doi: 10.1186/s40249-019-0544-6 (PMC6526597; doi:10.1186/s40249-019-0544-6)
Supplement: Supplementary file 1 — Multilingual abstracts in the five official working languages of the United Nations. (PDF 534 kb) [file 40249_2019_544_MOESM1_ESM.pdf]

Translation of the abstract into the five official working languages of the United Nations

وصمة العار والصرع في المناطق التي يتوطنها داء كلابية الذنب في أفريقيا: مراجعة وتوصيات من الفريق العامل المعني بالصرع المرتبط بداء كلابية الذنب

Neill, Julia Irani, Joseph Nelson Fodjo Siewe, Nono Denis, Catherine Abbo, Yasuaki Sato, ’Sarah O
Housseini Dolo, Maya Ronse, Alfred K Njamnshi and Robert Colebunders

خلاصة

الخلفية: في المناطق الموبوءة بمرض العمى النهري ، لا سيما في المناطق التي لديها برنامج لمكافحة داء كلابية الذنب دون المستوى الأمثل ، يلاحظ ارتفاع معدل انتشار الصرع. كل من داء كلابية الذنب والصرع ظروف وصم. في الفترة من 12 إلى 14 أكتوبر 2017، عقدت أول ورشة عمل دولية عن الصرع المرتبطة داء كلابية الذنب (OAE) في أنتويرب، بلجيكا وتأسس التحالف AEO. في هذه الورقة ، نقوم بمراجعة ما هو معروف عن وصمة العار المرتبطة بالصرع في المناطق الموبوءة بمرض العمى النهري ، ونقدم توصيات مجموعة عمل تحالف OAE حول الوصمة.

النص الرئيسي: بالنسبة إلى هذا الاستعراض النطاق ، أجريت عمليات بحث الأدب على قواعد البيانات الإلكترونية PubMed و Scopus و Science Direct باستخدام مصطلحات البحث "الصرع AND داء كلابية الذنب AND وصمة العار ". كما أجريت عمليات البحث اليدوية باستخدام الباحث العلمي من Google ، وتم تحديد ما مجموعه سبع ورقات تناولت وصمة العار المرتبطة بالصرع في منطقة مستوطنة لداء العمى النهري. نظرًا للعدد المحدود من الأبحاث المنشورة حول وصمة العار المرتبطة بالصرع في المناطق الموبوءة بمرض العمى النهري ، تتم مناقشة الأدبيات الأخرى ذات الصلة التي تصف الجوانب المهمة المتعلقة بالوصمة. يتبع العرض المواضيعي لمراجعة النطاق هذه رؤى أساسية حول الحواجز التي تحول دون التخفيف من العواقب الاجتماعية للوصمة في المناطق الموبوءة بشدة بداء كلابية الذنب ، والتي أنشأها خبراء خلال مجموعة العمل المعنية بالوصمة والتمييز في ورشة العمل الدولية الأولى حول AEO. هذه الموضوعات هي: ثغرات المعرفة ، المسببات المرضية المدركة ، الوصول إلى التعليم ، قيود الزواج ، الرفاه النفسي والاجتماعي ، العبء على مقدم الرعاية وسلوك العلاج. استنادًا إلى الأدبيات ومناقشات الخبراء خلال مجموعة عمل OAE حول الوصمة ، تصف هذه الورقة القضايا الهامة المتعلقة بالوصمة المرتبطة بالصرع في المناطق الموبوءة بداء كلابية الذنب وتوصي بالتدخلات اللازمة للحد من وصمة العار والتمييز من أجل تحسين الرفاهية النفسية - الأشخاص الذين يعانون من الصرع.

الاستنتاجات: تثقيف العاملين في مجال الرعاية الصحية والمجتمعات حول OAE ، وتعزيز برامج القضاء على داء كلابية الذنب ، وتقليل فجوة العلاج المضادة للصرع ، وتحسين رعاية الإصابات المرتبطة بالصرع ، وتحديد أولويات أبحاث الصرع الطريق إلى الأمام لتقليل وصمة العار المرتبطة بالصرع في المناطق الموبوءة بمرض العمى النهري.

Translated from English version into Arabic by Aalya Al-Beeshi, proofread by Aaminah Natha, through

TRANSLATORS WITHOUT BORDERS

非洲盘尾丝虫病流行地区的耻辱感和癫痫：文献综述和盘尾丝虫病相关癫痫工作组的建议

Sarah O’Neill, Julia Irani, Joseph Nelson Fodjo Siewe, Nono Denis, Catherine Abbo, Yasuaki Sato, Housseini Dolo, Maya Ronse, Alfred K Njamnshi and Robert Colebunders

摘要

引言：在盘尾丝虫病流行地区，特别是在盘尾丝虫病防治计划实施欠佳的地区，癫痫的发病率仍然较高。盘尾丝虫病和癫痫都会给患者带来耻辱感。第一届关于盘尾丝虫病相关性癫痫（OAE）的国际研讨会在比利时安特卫普举行（2017 年 10 月 12–14 日），会议期间成立了 OAE 联盟。本

文回顾了盘尾丝虫病流行地区癫痫相关耻辱感的知识，并提出了 OAE 联盟工作组关于耻辱感的建议。
主要内容：我们使用术语“epilepsy AND onchocerciasis AND stigma”在 PubMed、Scopus 和 Science Direct 电子数据库进行文献检索。此外，还在谷歌学术进行了手工搜索，共发现 7 篇论文涉及盘尾丝虫病流行地区的癫痫相关耻辱感的研究。由于盘尾丝虫病流行地区癫痫相关耻辱感的研究论文数量有限，因此，本文将其他与耻辱相关的重要文献纳入讨论。本综述遵循第一次 OAE 国际研讨会上的耻辱和歧视问题工作组所提出的一些关键见解，作了关于减少高度受影响的盘尾丝虫病流行地区因耻辱带来的社会后果的障碍的专题介绍。这些主题包括知识差距、感知疾病的病因、受教育机会、婚姻限制，心理社会福祉、护理者的负担和求医行为。根据文献和 OAE 关于耻辱感的工作组的专家讨论结果，本文描述了关于盘尾丝虫病流行地区癫痫相关耻辱的重要问题，并建议采取干预措施以减少对癫痫患者的羞辱感和所受到的歧视以提高其心理社会福祉。
结论：对医务人员和社区工作者开展关于 OAE 的健康教育，加强盘尾丝虫病消除计划，减少抗癫痫治疗差距，提高癫痫相关伤害护理水平，优先癫痫研究是在流行地区减少盘尾丝虫病癫痫相关耻辱的策略。

Translated from English version into Chinese by Xin-Yu Feng, edited by Pin Yang

CHINESE CENTER FOR DISEASE CONTROL AND PREVENTION
NATIONAL INSTITUTE OF PARASITIC DISEASES

Stigmatisation et épilepsie dans les régions d’onchocercose endémique en Afrique : revue et recommandations du groupe de travail sur l’épilepsie associée à l’onchocercose

Sarah O’Neill, Julia Irani, Joseph Nelson Fodjo Siewe, Nono Denis, Catherine Abbo, Yasuaki Sato, Housseini Dolo, Maya Ronse, Alfred K Njamnshi et Robert Colebunders

Résumé
Contexte: La prévalence de l’épilepsie est élevée dans les régions d’onchocercose endémique, en particulier celles qui ne sont pas couvertes par des programmes de lutte contre la maladie efficaces. Onchocercose et épilepsie sont deux maladies stigmatisantes. Le premier atelier international sur l’épilepsie associée à l’onchocercose (EAO) s’est tenu à Anvers, en Belgique, du 12 au 14 octobre 2017. Une alliance contre l’EAO a été créée à cette occasion. Dans le présent article, nous passons en revue les connaissances sur la stigmatisation de l’épilepsie dans les régions d’onchocercose endémique et présentons les recommandations du groupe de travail contre la stigmatisation de l’alliance contre l’EAO.
Discussion: Pour cette revue de cadrage, nous avons effectué des recherches dans les bases de données électroniques PubMed, Scopus et Science Direct en utilisant les termes de recherche «epilepsy AND onchocerciasis AND stigma». Nous avons également effectué des recherches manuelles dans Google Scholar. Nous avons identifié en tout sept articles traitant de la stigmatisation de l’épilepsie dans une région d’onchocercose endémique. En raison du petit nombre de recherches publiées sur cette question, nous avons inclus aussi d’autres publications pertinentes décrivant des aspects importants liés à la stigmatisation. La présentation thématique de cet examen de la portée retrace les principales idées sur les difficultés rencontrées dans l'atténuation des conséquences sociales de la stigmatisation dans les zones d’onchocercose fortement endémique, telles que les ont définies les experts du groupe de travail sur la stigmatisation et la discrimination au cours du premier atelier international sur l’EAO. Ces obstacles sont les suivants : manque de connaissances, croyances sur l’étiologie de la maladie, accès à l’éducation,

restrictions des mariages, bien-être psychosocial, charge pour le soignant et comportement de recherche de traitement. D'après la documentation et les discussions d'experts qui ont eu lieu dans le cadre du du groupe de travail de l'EAO sur la stigmatisation, notre article décrit des questions importantes concernant la stigmatisation de l'épilepsie dans les régions d'onchocercose endémique et recommande les interventions nécessaires pour réduire la stigmatisation et la discrimination afin d'améliorer le bien-être psychosocial des personnes épileptiques.

Conclusions: Eduquer les professionnels de la santé et les communautés au sujet de l'EAO, renforcer des programmes d'élimination de l'onchocercose, réduire l'écart dans le traitement de l'épilepsie, améliorer les soins des blessures liées à l'épilepsie et donnée la priorité à la recherche sur l'épilepsie sont autant de pistes pour réduire la stigmatisation associe à l'épilepsie dans les régions d'onchocercose endémique.

Translated from English version into French by Suzanne Assenat, proofread by Gwenaelle Le Jan-Moulart, through

TRANSLATORS WITHOUT BORDERS

Стигма и эпилепсия в эндемичных по онхоцеркозу регионах Африки: обзор и рекомендации рабочей группы по эпилепсии, обусловленной онхоцеркозом

Сара О'Нил (Sarah O'Neill), Джулия Ирани (Julia Irani), Джозеф Нельсон Фоджо Сьеве (Joseph Nelson Fodjo Siewe), Ноно Денис (Nono Denis), Кэтрин Аббо (Catherine Abbo), Ясуаки Сато (Yasuaki Sato), Хуссейни Доло (Housseini Dolo), Майя Ронсе (Maya Ronse), Альфред К. Ньямнши (Alfred K Njamnshi) и Роберт Коулбандерс (Robert Colebunders)

Реферат

Справочная информация: В областях, эндемичных по онхоцеркозу, особенно в районах с неоптимальной программой борьбы с онхоцеркозом, наблюдается высокая распространенность эпилепсии. И онхоцеркоз, и эпилепсия являются стигматизирующими состояниями. Первый международный семинар по эпилепсии, обусловленной онхоцеркозом (ЭОО), был проведен в Антверпене, Бельгия (12-14 октября 2017 г.), и во время этой встречи был создан альянс OAE. В этой статье мы рассмотрим то, что известно о стигме, связанной с эпилепсией, в эндемичных по онхоцеркозу регионах и представим рекомендации рабочей группы альянса ЭОО по стигме.

Основная часть: В этом обзорном анализе поиск литературы проводился в электронных базах данных PubMed, Scopus и Science Direct с использованием поисковых терминов «эпилепсия, онхоцеркоз, стигма». Ручной поиск также проводился с использованием Google Scholar, и в общей сложности было выявлено семь работ, посвященных стигме, связанной с эпилепсией, в эндемичной зоне онхоцеркоза. В связи с ограниченным количеством опубликованных научных работ по стигме, связанной с эпилепсией, в эндемичных по онхоцеркозу областях, обсуждается другая соответствующая литература, в которой описаны важные аспекты, связанные со стигмой. Тематическая постановка вопроса этого обзорного анализа следует ключевому пониманию барьеров для смягчения социальных последствий стигмы в сильно пораженных онхоцеркозом

эндемичных областях, которые были установлены экспертами в ходе рабочей группы по стигме и дискриминации на первом международном семинаре по ЭОО. Этими темами являются: пробелы в знаниях, этиология предполагаемых заболеваний, доступ к образованию, брачные ограничения, психосоциальное благополучие, бремя, ложащееся на лиц, осуществляющих уход, и поведение, при котором люди самостоятельно ищут лечения. Основываясь на литературных и экспертных дискуссиях в рамках рабочей группы ЭОО по стигме, этот документ описывает важные вопросы, касающиеся стигмы, связанной с эпилепсией, в эндемичных по онхоцеркозу регионах и рекомендует меры, необходимые для снижения стигмы и дискриминации, для улучшения психосоциального благосостояния людей с эпилепсией.
Выводы: Информирование работников здравоохранения и местных сообществ об ЭОО, усиление программ по ликвидации онхоцеркоза, сокращение разрыва в противоэпилептическом лечении, улучшение помощи при травмах, связанных с эпилепсией, и приоритизация исследований эпилепсии — это путь к снижению стигмы, связанной с эпилепсией в эндемичных по онхоцеркозу регионах.

Translated from English version into Russian by Michael Kovalevich, proofread by Michael Orlov, through

TRANSLATORS
WITHOUT BORDERS

Estigma y epilepsia en regiones de África en las que la oncocercosis es endémica: una revisión y recomendaciones del grupo de trabajo sobre la epilepsia relacionada con la oncocercosis

Sarah O'Neill, Julia Irani, Joseph Nelson Fodjo Siewe, Nono Denis, Catherine Abbo, Yasuaki Sato, Housseini Dolo, Maya Ronse, Alfred K Njamnshi y Robert Colebunders

Resumen

Antecedentes: en las zonas en las que la oncocercosis es endémica, especialmente en aquellas con un programa deficiente para el control de la oncocercosis, se observa una alta prevalencia de epilepsia. Tanto la oncocercosis como la epilepsia son enfermedades estigmatizantes. Del 12 al 14 de octubre de 2017, se celebró el primer taller internacional sobre epilepsia relacionada con la oncocercosis (OAE, por sus siglas en inglés) en Amberes, Bélgica, y durante esta reunión se estableció la alianza OAE. En el presente artículo, hacemos una revisión de lo que se sabe acerca del estigma relacionado con la epilepsia en las regiones en las que la oncocercosis es endémica y presentamos las recomendaciones del grupo de trabajo de la alianza OAE sobre el estigma.
Texto principal: para esta revisión sistemática exploratoria se realizaron búsquedas bibliográficas en las bases de datos electrónicas PubMed, Scopus y Science Direct utilizando los términos de búsqueda «epilepsia Y oncocercosis Y estigma». Asimismo, se llevaron a cabo búsquedas manuales utilizando Google Scholar y se identificó un total de siete artículos que abordaban el estigma relacionado con la epilepsia en una zona en la que la oncocercosis es endémica. Debido al número limitado de artículos de investigación publicados sobre el estigma relacionado con la epilepsia en zonas en las que la oncocercosis es endémica, se examinan otros documentos pertinentes que describen aspectos importantes relacionados

con el estigma. La presentación temática de esta revisión sistemática exploratoria sigue las ideas clave sobre los obstáculos para mitigar las consecuencias sociales del estigma en las zonas altamente afectadas en las que la oncocercosis es endémica, establecidas por los expertos durante el grupo de trabajo sobre el estigma y la discriminación en el primer taller internacional sobre OAE. Estos temas incluyen: brechas de conocimiento, etiología percibida de la enfermedad, acceso a la educación, restricciones matrimoniales, bienestar psicosocial, carga para los cuidadores y búsqueda de tratamiento. Sobre la base de la literatura y los debates de expertos durante el grupo de trabajo OAE sobre el estigma, el presente artículo describe cuestiones importantes relativas al estigma relacionado con la epilepsia en regiones en las que la oncocercosis es endémica y recomienda intervenciones necesarias para reducir el estigma y la discriminación a fin de mejorar el bienestar psicosocial de las personas que sufren de epilepsia.

Conclusiones: la forma de avanzar para reducir el estigma relacionado con la epilepsia en regiones en las que la oncocercosis es endémica es educar a los trabajadores sanitarios y a las comunidades sobre la OAE, fortalecer los programas de eliminación de la oncocercosis, reducir la brecha del tratamiento antiepiléptico, mejorar la atención de lesiones relacionadas con la epilepsia y priorizar la investigación de la epilepsia.

Translated from English version into Spanish by Mayra León, proofread by Natalia Victoria Gómez, through

TRANSLATORS WITHOUT BORDERS
